# FDG-PET/CT Avid Uptake of a Biopsy-Proven Aggressive Melanotic Schwannoma of the S2 Spinal Nerve Root

**DOI:** 10.1055/s-0044-1791694

**Published:** 2024-10-08

**Authors:** Mohammed A. Azab, Hamid Abdelma'aboud Mostafa, Oday Atallah

**Affiliations:** 1 Department of Neurosurgery, Cairo University, Cairo, Egypt; 2 Department of Neurosurgery, Hannover School of Medicine, Hannover, Germany

**Keywords:** melanocytic schwannoma, FDG-PET/CT, MMNST

## Abstract

Malignant melanotic nerve sheath tumors (MMNSTs), also known as a melanocytic schwannoma (MS), are a rare type of peripheral nerve sheath tumors including Schwann cells with melanocytic differentiation. Only a few cases of spinal MMNST have been reported in literature. Fluorine-18 fluorodeoxyglucose positron emission tomography/computed tomography ( ^18^ F-FDG-PET/CT) could be used to detect these lesions. A 70-year-old man with a 6-month history of backache was admitted to our hospital. PET/CT showed a paravertebral soft tissue mass along the spinal nerve at the S2 level with strong FDG uptake, and a nodule with increased FDG uptake in the right lobe of the left liver. A CT-guided biopsy of the S2 lesion was performed. The final diagnosis was spinal MS with hepatic metastasis. The patient received stereotactic body radiation therapy. Herein, we report the PET/CT findings of a case of MS with hepatic metastasis. FDG-PET/CT is helpful in the differential diagnosis of benign and malignant lesions although nonspecific.

## Introduction

Melanotic schwannoma is a very rare variant of schwannoma that represents approximately 1% of all primary peripheral nerve sheath tumors. 1 In 1932, Millar first described this pathology in which Schwan cells can produce melanin based on its common embryological origins with melanocytes. 1 2 It frequently affects people aged 30 to 40. 3 This type of nerve tumor expresses special pathological markers differentiating it from conventional melanoma such as S-100, leu-7, and vimentin. 4

F18-fluorodeoxyglucose positron emission tomography/computed axial tomography (18F-FDG-PET/CT) could be used as a tool for diagnosis of this tumor. On FDG-PET, schwannomas show a high tumor-to-background ratio. 5 About 22 schwannomas reported in literature showed avid FDG uptake. 6 However, there is a wide variability of the FDG uptake of schwannoma throughout the literature. In this case report, we report a high FDG uptake of a biopsy-proven spinal nerve melanocytic schwannoma (MS).

## Case Description

**Fig. 1:**
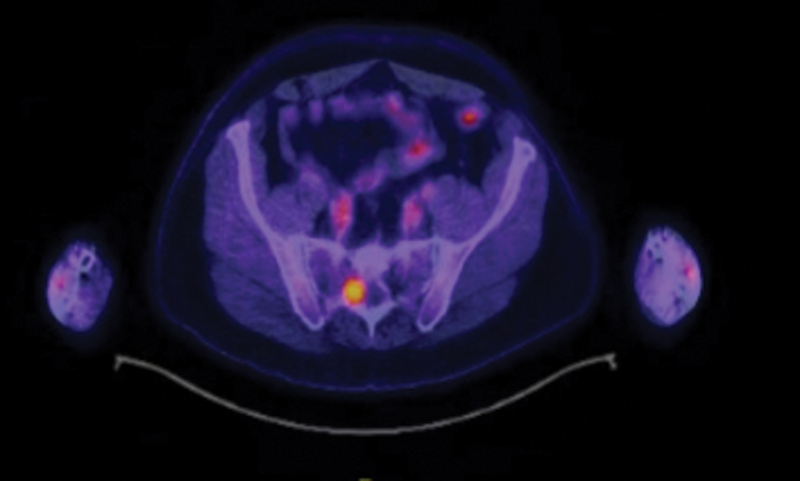
PET/CT image showed increased FDG uptake in the right S2 nerve root.

**Fig. 2:**
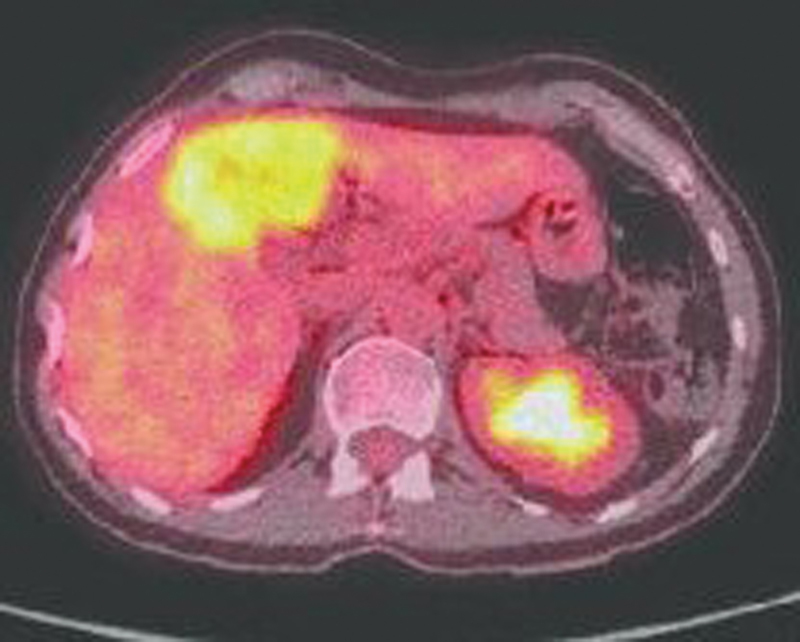
Positron emission tomography (PET) scan of the liver shows avid uptake in the right lobe of the liver.

**Fig. 3:**
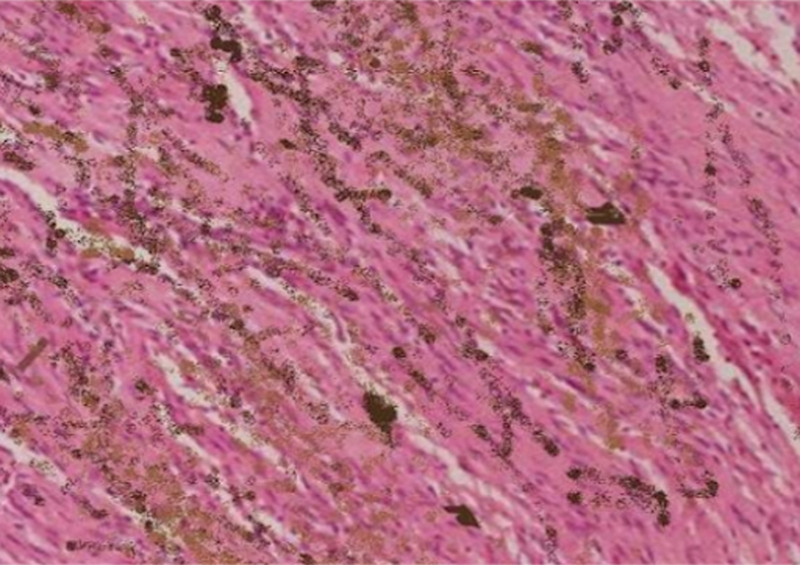
Hematoxylin and Eosin stained slide showing expansile, cell dense proliferation of neoplastic spindloid cells interspersed with neoplastic pigmented cells (200 magnification).

A 70-year-old man with a 6-month history of backache was admitted to our hospital. PET/CT showed a paravertebral soft tissue mass along the S2 spinal nerve with a strong FDG uptake (maximum standardized uptake value [SUVmax] 9.2) ( Fig. 1 ), and a nodule with increased FDG uptake in the right lobe of the liver ( Fig. 2 ). Tumor markers were in the normal range. A CT-guided biopsy of the S2 lesion was performed. Pathological examination revealed spindle and epithelioid cells arranged in interlacing fascicles and nests with accumulation of melanin in neoplastic cells and associated melanin containing macrophages ( Fig. 3 ). The final diagnosis was spinal MS with liver metastasis. The patient received stereotactic body radiation therapy and immunotherapy in the form of nivolumab and ipilimumab. The patient was followed up over 6 months with a regular PET scan and showed a reduction in the SUVmax value to 2.6. He experienced marked improvement of his backache.

## Discussion

MSs represent less than 1% of peripheral nerve sheath tumors and predominantly affect both genders equally. It commonly affects the cervical and upper thoracic spinal nerves, but can also arise in other locations such as the orbit, acoustic nerve, and the cerebellum. 1 2 7 Microscopically, they show bundle-shaped, interleaved, and wheel-shaped spindle cells with melanocytic pigments. There are many theories to explain MS pathogenesis, one of them is explained by the common developmental origin of melanocyte and Schwann cells. 3 Other possible differential diagnoses include neurofibroma, pigmented dermatofibrosarcoma, melanocytoma, and malignant melanoma. 5 MS stains positive for S-100, leu-7, HMB-45, and vimentin and negative for glial fibrillary acidic protein, epithelial membrane antigen, and creatine kinase. 8 magnetic resonance imaging (MRI) has a great role in MS diagnosis because of the paramagnetic feature of melanin. Conventional CT and MRI are potential tools for identifying peripheral nerve lesions and their relationship to nearby structures; however, they are not highly reliable to identify benign from malignant nerve sheath tumors. FDG-PET/CT is a potential tool in differentiating malignant from benign lesions, staging of malignant ones, evaluating the efficacy of chemotherapy and radiotherapy, and has prognostic implications. 6 9

Ferner et al reported that semiquantitative FDG-PET/CT analysis by calculating the SUVmax within a malignant peripheral nerve tumor had a statistically significant increase in SUVmax compared with benign lesions. 10 There was a remarkable difference in the SUV uptake between benign and malignant lesions (1.5 and 5.7, respectively). Moreover, the incremental increase in delayed uptake in PET scans could differentiate malignant lesions. 11 Chirindel et al used a late acquisition protocol (4 vs. 1 hour) to improve the diagnostic performance; however, it was not efficient for differentiating malignant peripheral nerve sheath tumor (MPNST) from benign peripheral nerve sheath tumors (BPNST). 12 Some authors considered that FDG-PET has limited value for identifying BPNST versus MPNST. 6 13

Schwannomas show heterogeneous SUVmax uptake. High uptake with SUV = 12, was evident in a malignant paravertebral schwannoma, while another false positive paravertebral schwannoma showed a high uptake of 6.7 for malignancy. 14 15 Beaulieu et al found no correlation between uptake and cellularity or proliferative index in schwannoma which makes PET scan ineffective in grading malignant schwannoma. 13 Assessment of SUV uptake should be carefully considered as it is affected by the heterogeneous consistency and shape of the lesion which complicates interpretation. 16 Overall, there is a wide variation in current literature regarding this issue, partly due to variations among scanners and scanning protocols. 16 17 18 However, the SUVmax threshold of ≥ 3.5 is commonly used as a marker of malignancy, although it may represent false positive results. 19 20 21 For the detection of MPNST in neurofibromatosis type 1 (NF1) patients, PET/CT is the most efficient imaging technique. 20 21 PET/CT imaging alone is not specific enough to diagnose MPNST, and a histopathological analysis remains the gold standard for optimal diagnosis. Brahmi et al evaluated the safety and efficacy of PET-guided biopsy of MPNSTs. They found that it is an effective procedure for diagnosis of NF1-related MPNST. 22

There are several postsurgical management strategies that include radiotherapy, stereotactic radiosurgery, chemotherapy, and immunotherapy. 2 9 23 Despite the benign behavior of schwannoma, MS showed unpredictable prognosis and tendency to metastasize. To the best of our knowledge, there are only five case reports of MS with hepatic metastasis ( Table 1 ). Shen et al reported a patient diagnosed with MS associated with liver metastasis by 18F-FDG-PET/CT and confirmed with biopsy. The patient was treated with six cycles of Endostar and temozolomide combined chemotherapy and survived for 1 year after initial diagnosis. 24 Haleem et al reported a similar patient with pain in S1 distribution diagnosed with schwannoma by MRI and after 12 months confirmed by biopsy and 18F-FDG-PET/CT. The FDG-PET SUVmax was 3.6 in this patient, while in our patient, the SUVmax uptake was 11.5. 4 In our patient, we did not rely completely on the PET scan results, we biopsied the lesion. There is a wide range of the FDG-PET uptake values of schwannoma, with some studies showing high uptake while others showing a lower uptake. Therefore, we favored biopsying this lesion.

**Table 1:** Summary of cases with liver metastases

Study id	Case no.	Gender	Age (y)	Primary site	Treatment	Follow-up (mo)/Outcome
Vallat-Decouvelaere et al (1999) 7	1	F	45	T6	Surgery	36/DOD
Torres-Mora et al (2014) 1	2	F	23	L4	Unknown	44/AWD
Torres-Mora et al (2014) 1	3	M	47	L3-L4	Unknown	5/DOD
Torres-Mora et al (2014) 1	4	F	67	T10	Unknown	10/DOD
Shen et al (2021) 24	5	F	29	L2-L3	Chemotherapy	12/AWD

Abbreviations: AWD, alive with disease; DOD, dead of disease; F, female; L, lumbar spine; M, male; T, thoracic spine.

## Conclusion

Unlike common schwannomas, MS is a rare type with metastatic tendency and a high recurrence rate. It shows avid uptake on FDG-PET scan as we report in this patient. Stereotactic radiosurgery may be a possible treatment option combined with immunotherapy.
